# From Fairies to Giants: Untangling the Effect of Body Size, Phylogeny, and Ecology on Vertebral Bone Microstructure of Xenarthran Mammals

**DOI:** 10.1093/iob/obad002

**Published:** 2023-01-16

**Authors:** E H Zack, S M Smith, K D Angielczyk

**Affiliations:** Department of Organismal Biology and Anatomy, University of Chicago, Chicago, IL 60637, USA; Negaunee Integrative Research Center, Field Museum of Natural History, Chicago, IL 60605, USA; Negaunee Integrative Research Center, Field Museum of Natural History, Chicago, IL 60605, USA

## Abstract

Trabecular bone is a spongy bone tissue that serves as a scaffolding-like support inside many skeletal elements. Previous research found allometric variation in some aspects of trabecular bone architecture (TBA) and bone microstructure, whereas others scale isometrically. However, most of these studies examined very wide size and phylogenetic ranges or focused exclusively on primates or lab mice. We examined the impact of body size on TBA across a smaller size range in the mammalian clade Xenarthra (sloths, armadillos, and anteaters). We µCT-scanned the last six presacral vertebrae of 23 xenarthran specimens (body mass 120 g–35 kg). We collected ten gross-morphology measurements and seven TBA metrics and analyzed them using phylogenetic and nonphylogenetic methods. Most metrics had similar allometries to previous work. However, because ecology and phylogeny align closely in Xenarthra, the phylogenetic methods likely removed some covariance due to ecology; clarifying the impact of ecology on TBA in xenarthrans requires further work. Regressions for Folivora had high *P*-values and low *R*-squared values, indicating that the extant sloth sample either is too limited to determine patterns or that the unique way sloths load their vertebral columns causes unusually high TBA variation. The southern three-banded armadillo sits far below the regression lines, which may be related to its ability to roll into a ball. Body size, phylogeny, and ecology impact xenarthran TBA, but parsing these effects is highly complex.

## Introduction

The impact of body size on skeletal morphology and evolution is a classic question in evolutionary biology and has been examined in a multitude of systems ranging from sauropods (Sander et al. 2011) and cetaceans (Gillet et al. 2019) to miniature frogs (Yeh 2002) and more (Hanken and Wake 1993; Smith et al. 2010; Smith and Lyons 2011; Field et al. 2013; Smith et al. 2018). The interaction between ecology and morphology is another perennial topic and forms the basis of functional morphology (Wainwright 1994; Thomason 1997; Ferry-Graham et al. 2002). However, despite the proliferation of research on body size, ecology, and morphology, work on the impact of body size and ecology on trabecular bone is much rarer (Fajardo et al. 2013; Mielke et al. 2018; Amson and Bibi 2021; Smith and Angielczyk 2022).

Trabecular bone is the spongy bone tissue found in the ends of vertebrate long bones, vertebral centra, ribs, and facial bones, where it serves as a scaffolding-like support to strengthen bones against forces acting on the skeleton (Wolff 1893; Kivell 2016). Trabecular bone remodels based on the direction and magnitude of *in vivo* forces according to the theory of bone functional adaptation, which states that the rod- and plate-like trabeculae follow the principal force trajectories generated from external loads (Wolff 1893; Cowin 1986; Huiskes et al. 2000; Ruff et al. 2006; Sugiyama et al. 2010; Barak et al. 2011; Kivell 2016; Willie et al. 2020). Because trabecular bone architecture (TBA) changes as force regime changes, and different bones are exposed to different forces, TBA properties differ based on the specific taxa and skeletal elements examined. Using a sample of ecologically diverse mammals from across the clade, Amson and Bibi (2021) found that vertebral TBA is much more affected by body size than humeral TBA. Furthermore, body size is a stronger determinant of TBA in vertebrae than ecology (Amson and Bibi 2021). However, these patterns were more complicated for certain ecologies, such as fully subterranean and fully aquatic. The detailed impacts of ecology, body size, and phylogeny on different aspects of TBA within smaller phylogenetic groups are still unresolved.

Parsing the influence of body size, ecology, and phylogeny on trabecular bone is complicated. However, the effect of body size on TBA has been previously examined in birds, reptiles, mammals, rodents, primates, and humans, and many aspects of TBA scale allometrically (Swartz et al. 1998; Doube et al. 2011; Barak et al. 2013; Fajardo et al. 2013; Ryan and Shaw 2013; Mielke et al. 2018; Wysocki and Tseng 2018; Plasse et al. 2019; Saers et al. 2019; Alfieri et al. 2021; Amson and Bibi 2021). Trabeculae have a minimum thickness of about 50 µm and a maximum of 460 µm, resulting from the mechanical limitations of osteoclasts and osteocytes, respectively (Lozupone and Favia 1990; Mullender and Huiskes 1995; Barak et al. 2013; Ryan and Shaw 2013), and trabecular thickness (Tb.Th) scales with negative allometry with body size (Barak et al. 2013; Ryan and Shaw 2013). In most previous work, bone volume fraction (BV.TV) has not been correlated with body size, but the arrangement of bone tissue has been shown to change as body size increases (Barak et al. 2013). Smaller animals, specifically mice and rats in laboratory settings, have relatively thicker trabeculae with larger spaces between them, whereas larger animals (humans) have relatively thinner trabeculae with less space between them (Barak et al. 2013). Trabecular number (Tb.N), therefore, scales with strong negative allometry (Ryan and Shaw 2013). All metrics concerning the orientation of trabeculae do not scale with size as they are primarily impacted by the direction and not magnitude of forces (Barak et al. 2011, 2013; Doube et al. 2011; Ryan and Shaw 2013). Connectivity density (Conn.D) scales inversely with size—trabeculae have fewer connections per unit volume as body size increases (Doube et al. 2011; Ryan and Shaw 2013).

Xenarthra is a clade of mammals that includes anteaters, armadillos, sloths, and their extinct relatives. Because they spent their evolutionary history isolated in the Americas, xenarthrans are both phylogenetically and morphologically disparate from other placental mammal groups (Gaudin 2003; Delsuc et al. 2019; Presslee et al. 2019; Varela et al. 2019). The clade is characterized by a low metabolic rate and body temperature, their absent or enamel-less teeth, their heavily ossified synsacrum, and the xenarthrous accessory articular process on their lumbar vertebrae (Gaudin 2003). The presence of the xenarthrous process on the postdiaphragmatic vertebrae of xenarthrans has been examined using a wide variety of methods (Gaudin and Biewener 1992; Galliari et al. 2010; Oliver et al. 2016; Hautier et al. 2018; Oliver et al. 2021; Miranda et al. 2022). This extra articulation point for posterior thoracic and lumbar vertebrae stiffens the vertebral column, which is thought to facilitate digging behavior by stiffening the trunk while allowing the animal to use its forelimbs for digging (Gaudin and Biewener 1992; Oliver et al. 2016). Other work has suggested that the morphology of the thoracolumbar region of the spinal column is strongly correlated to locomotor ecology, especially in arboreal and fossorial mammals (Jones et al. 2018). Because of this correlation and the importance of lumbar vertebrae in the history of this clade, we chose to examine TBA of lumbar vertebrae of Xenarthra.

**Fig. 1:**
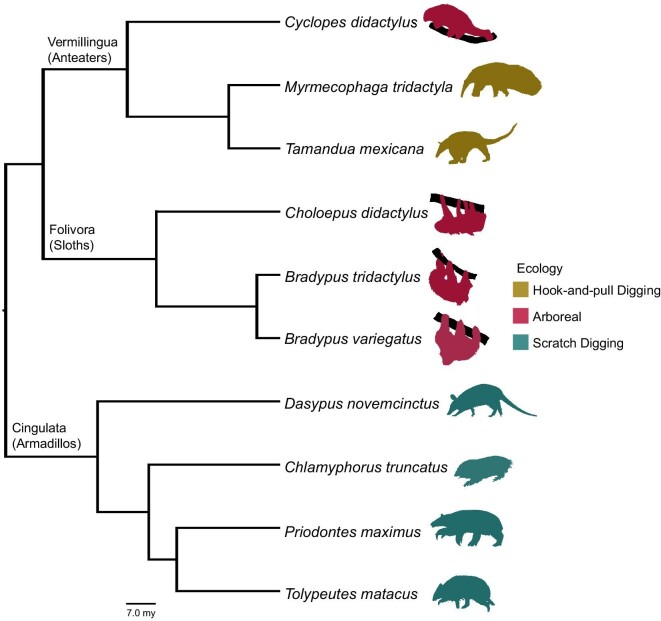
Phylogeny of xenarthran species included in this work with silhouettes colored by ecology. Silhouettes not to scale. my, million years.

Xenarthran body size variation is particularly interesting in the context of TBA: the extinct diversity of Xenarthra includes giant sloths the size of elephants and glyptodonts the size of small cars. However, extant xenarthran diversity ranges from *Myrmecophaga tridactyla* (120 kg) to *Chlamyphorus truncatus* (35 g) (Figs. S1 and S2) (Jones et al. 2009). The size ranges of both extant armadillos and extant anteaters are as large as the entire extant xenarthran range, but the extant sloth size range is relatively small in comparison. Scaling in the trabecular bone of xenarthrans has rarely been examined previously, but the superorder offers a phylogenetically conserved and ecologically diverse group of mammals in which to examine the impact of body size, ecology, and phylogeny on TBA (Fig. 1) (Amson et al. 2017; Alfieri et al. 2021). Modern xenarthrans provide an opportunity to examine the details of scaling patterns across a more limited size range and phylogenetic scale than in mammal-wide datasets.

A coincidence of the limited extant diversity of xenarthrans when compared to their extinct diversity is that phylogeny and ecology align almost exactly within the clade (Fig. 1). All members of Cingulata are scratch diggers, most Vermillingua are hook-and-pull diggers, and all extant members of Folivora are suspensorial arborealists (Rood 1970; Ramsey 1978; Shaw et al. 1987; Smith 2007; Hayssen 2009, 2010, 2011; Navarrette and Ortega 2010; Hayssen et al. 2012; Carter et al. 2016). This correlation makes untangling the impacts of ecology and phylogeny on TBA within the group particularly challenging. Therefore, an additional goal of this work was to examine the degree to which we could successfully partition phylogenetic and ecological variance when comparing results from phylogenetic and nonphylogenetic statistical methods. Currently, phylogenetic comparative methods (PCMs, such as phylogenetic generalized least squares [PGLS] and phylogenetic flexible discriminant analysis [pFDA]) assume that traits that happen to be codistributed are highly correlated. PCMs are unable to parse whether two traits are correlated if both traits examined happen to only be present in one derived clade (Uyeda et al. 2018, see Case Study 3). As shown by Uyeda et al. in 2018, these codistributed traits are not always evolutionarily correlated, and if used in this scenario, then current PCMs can be misleading (Uyeda et al. 2018). Using both phylogenetic and nonphylogenetic statistical methods will allow us to compare a method that assumes that phylogeny has no effect (nonphylogenetic methods) and a method that assumes that similarity between closely related organisms is due to their relatedness rather than to ecological convergences (phylogenetic methods) (Revell 2010; Ryan and Shaw 2013; Uyeda et al. 2018). Although this interpretation is a slight oversimplification due to the alignment of ecology and phylogeny in Xenarthra, using results from each method as the extremes of possible effects of ecology and phylogeny will allow us to better understand their impacts on the TBA of xenarthrans. We expect the true impact to be somewhere between these two extremes.

We predict that the allometric scaling of TBA in digging genera will match that of other mammal groups (Doube et al. 2011; Barak et al. 2013), but that metrics for suspensory genera will have a very weak or no scaling relationship. Additionally, because ecology and phylogeny overlap so closely in Xenarthra, we predict that common phylogenetic comparative methods may have difficulty correctly partitioning covariance due to phylogeny and covariance due to ecology.

## Methods

### Specimen choice and measurement

**Fig. 2:**
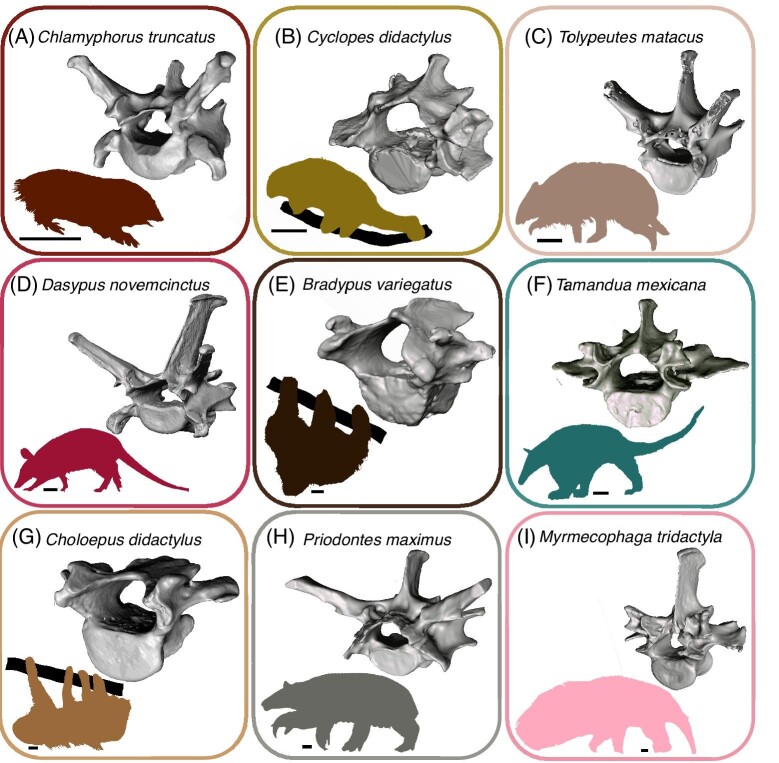
3D renderings of a representative first lumbar vertebra (L01) of each genus with silhouettes of an individual from each genus. Scale bars are 5 cm. (A) L01 of FMNH 105031 *C. truncatus*; (B) L01 of FMNH 69971 *Cy. didactylus*; (C) L01 of FMNH 28342 *T. matacus*; (D) L01 of FMNH 60493 *D. novemcinctus*; (E) Mesh of L01 of FMNH 68919 *B. variegatus*; (F) L01 of FMNH 18835 *T. mexicana*; (G) L01 of FMNH 95448 *Ch. didactylus*; (H) L01 of FMNH 25271 *P. maximus*; (I) L01 of FMNH 28309 *M. tridactyla*.

We chose 23 xenarthran specimens from the Field Museum of Natural History (FMNH) Mammalogy collections to undergo X-ray micro-computed tomography (μCT) scanning at the University of Chicago's PaleoCT facility (https://luo-lab.uchicago.edu/paleoCT.html). These specimens represent ten species in nine genera, span all three major xenarthran clades, and encompass much of the group's modern ecomorphological diversity and disparity (Table 1). Because TBA is affected by *in vivo* forces and development, we used only adult specimens (determined by fusion of the cranial sutures and long bone epiphyses) with full vertebral columns (Gaudin and Biewener 1992; Hautier et al. 2018; Oliver et al. 2016, 2021). To mitigate the impacts of captivity, we selected only specimens caught in the wild (Zack et al. 2021). Among the specimens selected, the number of lumbar vertebrae varied from two to five (Table 1), so we analyzed the last six presacral vertebrae to include all lumbar vertebrae across all specimens and standardize across specimens (Fig. 2).

**Table 1:** Specimen and taxa choice and categorization

Taxon	Size class (estimated mass [g])	Ecology	Number of specimens	Thoracic vertebrae (number [mean])	Lumbar vertebrae (number)	Scan resolutions (µm)
*Bradypus tridactylus*	Medium (4136.36)	Arboreal	1	15 (15)	3	46.833
*Bradypus variegatus*	Medium (4375.80)	Arboreal	3	16–17 (16)	3	41.997–45.906
*Chlamyphorus truncatus*	Small (85.53)	Scratch digging	4	11–12 (12)	3	22.101–24.243
*Choloepus didactylus*	Medium (6646.50)	Arboreal	2	23 (23)	3–4	49.589–56.169
*Cyclopes didactylus*	Small (263.95)	Arboreal	3	15–16 (15)	2	25.031–26.123
*Dasypus novemcinctus*	Medium (3949.01)	Scratch digging	3	10 (10)	5	41.61–42.931
*Myrmecophaga tridactyla*	Large (29531.83)	Hook-and-pull digging	2	15–16 (16)	2	93.178–96.551
*Priodontes maximus*	Large (40641.089)	Scratch digging	2	12–13 (13)	2	73.771–76.226
*Tamandua mexicana*	Medium (4178.51)	Hook-and-pull digging	3	16–17 (17)	2–3	44.643–46.692
*Tolypeutes matacus*	Small (1303.47)	Scratch digging	1	12 (12)	3	27.465

**Fig. 3:**
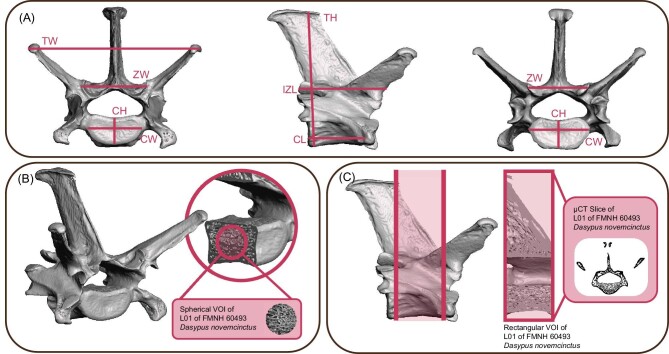
(A) Vertebral measurements shown on 3D renderings of a representative vertebra (L01 of FMNH 60493 *D. novemcinctus*) in cranial, right lateral, and caudal views. (B) 3D rendering of a representative vertebra (L01 of FMNH 60493 *D. novemcinctus*) with inset depicting the VOI used in TBA analyses as described in the text. (C) 3D rendering of a representative vertebra (L01 of FMNH 60493 *D. novemcinctus*) with VOI used in the analyses of GC and CSA shaded in pink. The inset depicts the VOI and a characteristic, binarized μCT slice. CH, centrum height; CL, centrum length; CW, centrum width; L01, first lumbar vertebra; TH, total height; TW, total width; ZW, zygapophyseal width.

As a proxy for body size, we took ten gross morphology measurements on each vertebra (Fig. 3A). When close articulation of the vertebrae prevented measurement of the physical specimens, we measured 3D meshes of the vertebrae in Autodesk MeshMixer or ORS Dragonfly 2020.1 (Object Research Systems 2019, Autodesk 2020). Using linear regressions of each measurement against body mass data found in PanTHERIA (Jones et al. 2009), we determined that inter-zygapophyseal length (IZL) was the vertebral dimension that most closely scaled with body size (Figs. 3A and 5A). We performed a generalized least squares (GLS) regression for the IZL of each vertebral position to determine the most accurate proxy for body mass and found that the IZL of the third vertebral position in our sample (ps3) had the highest *R*-squared value (Table S1). We therefore used IZL of ps3 as the proxy for body size in further analyses.

### Scanning and TBA analysis

We scanned all specimens using the 240 kV tube of the PaleoCT lab's GE v|tome|x μCT scanner at resolutions from 22.101 µm to 96.551 µm (average resolution 45.432 µm), depending on specimen size (Table 1). We reconstructed the scans in GE phoenix datos|x and aligned and cropped the reconstructed scans using VGStudio (Volume Graphics 2019). Following the method of Zack et al. (2021), we isolated prism-shaped volumes of trabecular bone from the centrum of each individual vertebra (Fig. 3B) (Smith and Angielczyk 2020; Zack et al. 2021). We determined the size and location of the prism by orienting the vertebra in cranial view, selecting the largest 2D square area of trabecular bone at the dorsoventrally and mediolaterally narrowest point of the centrum, and extending that square cranially and caudally to the edge of the cortical bone of the epiphyses (Fig. 3B). This volume of interest (VOI) included the largest possible volume of continuous trabecular bone with a standardized shape, no matter the shape or size of the vertebra (Fig. 3B).

**Fig. 4:**
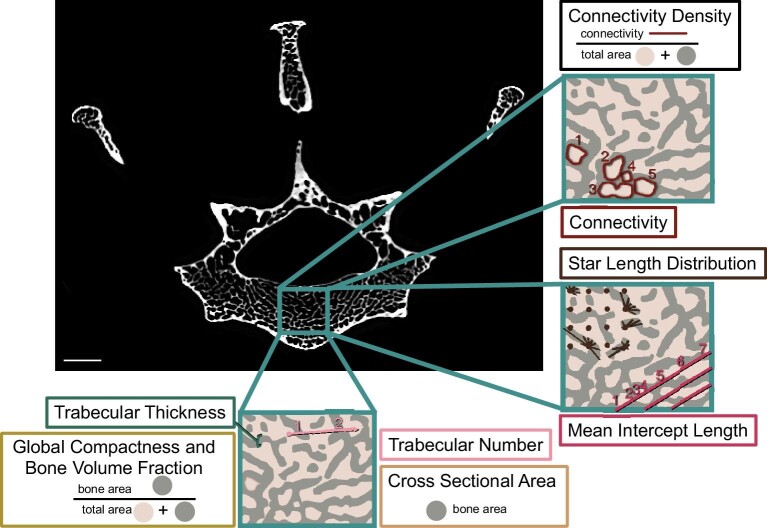
Representative μCT slice of T10 of FMNH 60493 (*D. novemcinctus*). Teal boxes indicate the VOI. The inset regions of interest include trabecular bone (gray) and intertrabecular space (light brown). Connectivity is calculated by counting the number of closed (circles) in a VOI as shown in dark red. Conn.D is connectivity per unit volume, as shown in black. Star length distribution is calculated by the method shown in dark brown. Mean intercept length is calculated as depicted in bright pink. Tb.N is calculated by the method approximated using the light pink arrow. Tb.Th is indicated by the green lines. The calculations used for BV.TV, GC, and CSA are shown in the yellow and tan boxes. Scale bar = 2 mm. T, thoracic. Adapted from Zack et al. (2021).

Using the “Threshold” tool in FIJI (Schindelin et al. 2012), we converted the VOI into a binary image stack in which we maximized bone sampling and minimized sampling of nonbone pixels (Schindelin et al. 2012; Amson et al. 2017). We analyzed the binary image stack in Quant3D using a user-defined threshold of 127–255 as required by the software to define the binary colors; 2049 uniform rotations; dense vectors and random rotations turned on; omitted side intersecting paths turned on; and star volume and star length distributions (SLDs) calculated with 2000 points (Ketcham and Ryan 2004; Ketcham 2005). We used the largest centered sphere that would fit within the rectangular VOI as the volume of interest for these calculations to eliminate edge-related artifacts (Fig. 3B). Quant3D outputs several values for analysis, including BV.TV, Tb.N, mean Tb.Th, and two measures of trabecular anisotropy (directionality): mean intercept length and SLD (Fig. 4). SLD is calculated by measuring the length of trajectories from where points on a randomly translated point grid are within the trabeculae, as shown in dark brown (Fig. 4) (Vesterby et al. 1989). We used the mean intercept length method for measuring degree of anisotropy (DA); mean intercept length is the number of places where the lines of a randomly translated linear grid intersect with the interface between trabecular bone and intertrabecular space (in bright pink) (Fig. 4) (Odgaard 1997). We calculated connectivity and Conn.D from the VOIs using the BoneJ macro in FIJI (Fig. 4) (Doube et al. 2010; Schindelin et al. 2012). Connectivity is calculated as the number of closed curves in a VOI, and approximates the number of connected trabecular elements in a volume of bone (Odgaard and Gundersen 1993; Toriwaki and Yonekura 2002). Conn.D is the number of connected trabecular elements per unit of volume (Odgaard 1997). We excluded any specimens with a connectivity of less than 40, a threshold chosen because of the small maximum possible VOI size obtainable in the smallest organisms in our dataset (Mielke et al. 2018). Together, the metrics we collected quantify important characteristics of the trabecular bone related to strength and trabecular direction (Silva and Gibson 1997; Ulrich et al. 1999).

To verify the accuracy of our measurements, we calculated relative resolution (scan resolution/Tb.Th, yielding the mean width of a trabecular strut in pixels). All specimens had a relative resolution of greater than 2.5 when measured using Quant3D. This is lower than the relative resolution usually recommended for this type of analysis (Sode et al. 2008; Kivell et al. 2011), but those recommendations used BoneJ to calculate Tb.Th (Smith and Angielczyk 2022). Quant3D yields consistently lower Tb.Th values compared to BoneJ due to the different methods of calculation (Ketcham and Ryan 2004; Doube et al. 2010). However, previous work (Smith and Angielczyk 2022) shows that a relative resolution of 2.5 px/tb in Quant3D is roughly equivalent to 4.4 px/tb in BoneJ, suggesting that our minimum relative resolution is sufficient for accurate measurements.

To analyze bone microstructure beyond TBA, we measured global compactness (GC) and cross-sectional area (CSA) for each vertebra using Amson's (2019) method (Figs. 3C and 4). We segmented out entire vertebrae and binarized them using the same threshold as used to binarize the centrum VOI. Using the FIJI macro written by Amson (2019), we calculated CSA and GC for each vertebra including only the slices where the vertebral foramen was completely surrounded by bone (Fig. 3C) (Doube et al. 2010; Schindelin et al. 2012; Amson 2019). CSA measures the area of bone in each slice, including both cortical bone and trabecular bone. GC measures the CSA of the bone divided by the total CSA (Fig. 4) (Amson 2019).

### Regressions and discriminant function analyses

For all regressions and flexible discriminant analyses (FDAs), we chose to use both phylogenetic and nonphylogenetic methods because ecology and phylogeny are closely aligned in extant xenarthrans, likely making it difficult to cleanly isolate phylogenetic and ecological signals (Fig. 1) (Zack et al. 2021). Using both phylogenetic and nonphylogenetic methods will allow us to directly compare an analysis that assumes that phylogeny has no effect to an analysis that assumes the similarity of organisms is due to their relatedness rather than to convergence (Revell 2010). The phylogenetic regressions we used do not allow for more than one specimen from each species, so we had to use the average of each. Because of this, we also calculated regressions using the species means for each vertebral position. If there is no impact of using the species means, then we would expect the species mean regressions to be exactly the same as the nonphylogenetic specimen-based regressions. Comparing these three methods will allow us to better understand how the inclusion of phylogenetic information is affecting the slope estimation in these regressions.

**Fig. 5:**
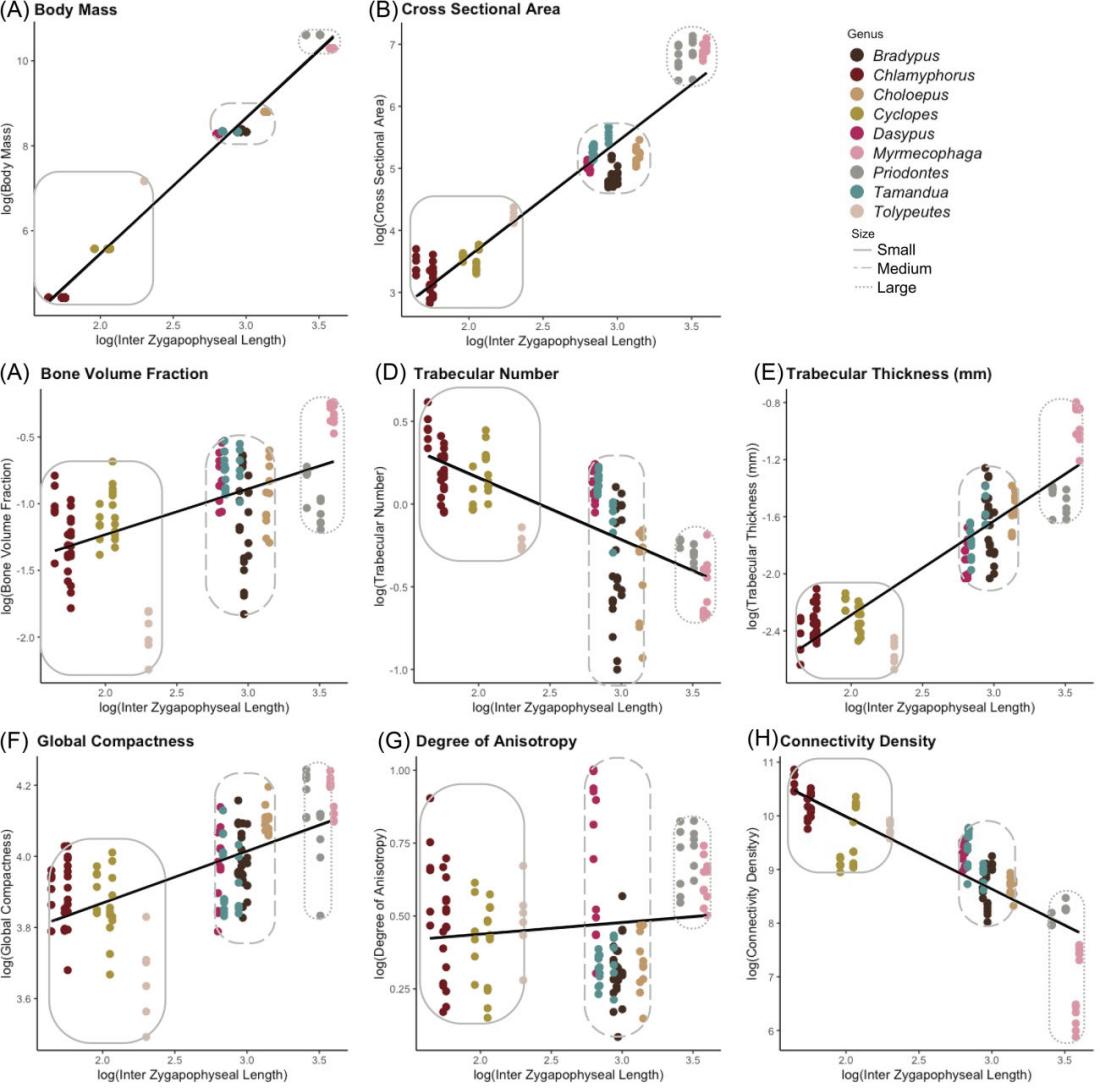
Log10 corrected GLS regressions with size classes depicted by light gray rounded rectangles. Point color corresponds to genus, and line type of the outline of rectangles corresponds to size class.

To statistically analyze the impact of body size on each bone microstructure metric, we used GLS regressions on log-transformed metrics for the entire dataset (Table 2, Fig. 5). We additionally calculated the linear regressions for species averages (Table S3, Fig. S3). BV.TV and GC are ratios and are therefore unitless with an isometric slope of 0. Tb.N is measured in numbers/mm, so the isometric slope is −1. Tb.Th is a length value with an isometric slope of 1; CSA is a measure of area with an isometric slope of 2. Isometric slope for Conn.D is −3 because it is measured in numbers/mm^3^ (see Mielke et al. 2018 for further explanation of isometry for these metrics; Plasse et al. 2019). We also calculated confidence intervals (CIs) for all regressions using the confint function in R (Team RC 2022). A slope was considered allometric if the isometric slope fell outside the CI. We calculated the regressions for each of the three clades examined (Cingulata [armadillos], Vermillingua [anteaters], and Folivora [sloths]) and for each of the ecologies (arboreal, hook-and-pull digging, and scratch digging). We then compared the slopes using the standardized major axis estimation and testing function in the smatr package in R (Tables 3 and 4) (Warton et al. 2012; Team RC 2022). We also used PGLS regressions to determine the impact of phylogenetic covariance on the scaling of TBA metrics (Table 2, S2). We pruned the time-scaled tree of Gibb et al. (2015) to include only the species in our dataset. We prepared the data by calculating the species means of each metric by vertebral position. We used the GLS function and corBrownian in the R package ape to calculate the PGLS of each metric (Table 2, S2) (Paradis and Schliep 2019). We calculated Blomberg's *K* using the R package phytools to further analyze the phylogenetic signal of each variable (Revell 2012). Because we analyzed each vertebral position separately, we used the average of the PGLS regressions of each metric along the vertebral column to compare to the individual linear regressions.

**Table 2:** Linear regression and phylogenetic regression. Statistical significance indicated by*

Formula	Isometric slope	GLS slope	GLS confidence interval (lower–upper)	GLS allometry	Average PGLS slope (range)	Average PGLS confidence interval (lower–upper)	PGLS allometry	GLS slope *P*-value (ɑ < 0.05)	Average PGLS slope *P*-value (ɑ < 0.05)	GLS R squared
log(BV.TV) ∼ log(IZL)	0	0.33	0.23–0.42	+	0.43 (0.35–0.50)	−0.22 to 1.08	0	<<0.001*	0.174	0.267
log(Tb.N) ∼ log(IZL)	−1	−0.28	−0.44 to −0.31	+	−0.39 (−0.44 to −0.32)	−0.81 to 0.03	+	<<0.001*	0.087	0.491
log(Tb.Th) ∼ log(IZL)	1	0.66	0.60–0.72	–	0.74 (0.69–0.77)	0.41–1.08	0	<<0.001*	0.003*	0.795
log(GC) ∼ log(IZL)	0	0.14	0.11–0.17	+	0.21 (0.17–0.26)	0.039–0.38	+	<<0.001*	0.025*	0.395
log(CSA) ∼ log(IZL)	2	1.84	1.74–1.95	–	2.04 (1.95–2.12)	1.69–2.39	0	<<0.001*	<<0.001*	0.901
log(DA) ∼ log(IZL)	0	0.04	−0.01 to 0.10	0	0.16 (0.10– 0.22)	−0.04 to 0.36	0	0.151	0.120	0.008
log(Conn.D) ∼ log(IZL)	−3	−1.35	−1.51 to −1.20	+	−1.67 (−1.75 to −1.61)	−2.51 to −0.83	+	<<0.001*	<<0.001*	0.691
log(mass) ∼ log(IZL)	3	3.20	3.12–3.29	+	3.16 (3.16)	2.78–3.54	0	<<0.001*	<<0.001*	0.978

**Table 3:** Equality of slopes taxa. Statistical significance indicated by*

Formula	Taxon	Slope	*R*-squared	Regression *P*-value (ɑ < 0.05)	Allometry	Equality	Equality *P*-value (ɑ < 0.05)
log(BV.TV) ∼ log(IZL)	Cingulata	0.231	0.182	0.001*	+	Slopes are not equal.	<<0.001*
	Vermillingua	0.490	0.812	<<0.001*	+		
	Folivora	0.820	0.040	0.255	0		
log(Tb.N) ∼ log(IZL)	Cingulata	−0.277	0.559	<<0.001*	+	Slopes are not equal.	<<0.001*
	Vermillingua	−0.403	0.622	<<0.001 *	+		
	Folivora	−0.086	0.001	0.894	0		
log(Tb.Th) ∼ log(IZL)	Cingulata	0.497	0.810	<<0.001*	–	Slopes are not equal.	<<0.001*
	Vermillingua	0.843	0.929	<<0.001*	–		
	Folivora	0.200	0.007	0.649	0		
log(GC) ∼ log(IZL)	Cingulata	0.121	0.292	<<0.001*	0	Slopes are not equal.	<<0.001*
	Vermillingua	0.172	0.557	<<0.001*	+		
	Folivora	0.597	0.283	0.001*	+		
log(CSA) ∼ log(IZL)	Cingulata	1.952	0.960	<<0.001*	0	Slopes are not equal.	0.008*
	Vermillingua	2.137	0.990	<<0.001*	+		
	Folivora	2.185	0.616	<<0.001*	0		
log(DA) ∼ log(IZL)	Cingulata	0.132	0.198	0.001*	+	Slopes are not equal.	<<0.001*
	Vermillingua	0.109	0.195	0.002*	+		
	Folivora	0.209	0.030	0.330	0		
log(Conn.D) ∼ log(IZL)	Cingulata	−1.202	0.920	<<0.001*	++	Slopes are not equal.	<<0.001*
	Vermillingua	−1.549	0.600	<<0.001*	++		
	Folivora	0.108	0.001	0.873	++		
log(mass) ∼ log(IZL)	Cingulata	3.530	0.990	<<0.001*	+	Slopes are not equal.	<<0.001*
	Vermillingua	2.956	0.993	<<0.001*	0		
	Folivora	2.331	0.938	<<0.001*	–		

**Table 4:** Equality of slopes ecology. Statistical significance indicated by*

Formula	Ecology	Slope	*R*-squared	Regression *P*-value (ɑ < 0.05)	Allometry	Equality	Equality *P*-value (ɑ < 0.05)
log(BV.TV) ∼ log(IZL)	Arboreal	0.055	0.008	0.520	0	Slopes are equal.	0.494
	Hook-and-pull digging	0.599	0.772	<<0.001*	+		
	Scratch digging	0.231	0.182	0.002*	+		
log(Tb.N) ∼ log(IZL)	Arboreal	−0.517	0.484	<<0.001*	+	Slopes are not equal.	<<0.001*
	Hook-and-pull digging	0.830	0.833	<<0.001*	+		
	Scratch digging	−0.277	0.559	<<0.001*	+		
log(Tb.Th) ∼ log(IZL)	Arboreal	0.646	0.750	<<0.001*	–	Slopes are not equal.	<<0.001*
	Hook-and-pull digging	1.082	0.908	<<0.001*	0		
	Scratch digging	0.497	0.810	<<0.001*	–		
log(GC) ∼ log(IZL)	Arboreal	0.159	0.436	<<0.001*	+	Slopes are not equal.	0.004948*
	Hook-and-pull digging	0.300	0.618	<<0.001*	+		
	Scratch digging	0.121	0.292	<<0.001*	+		
log(CSA) ∼ log(IZL)	Arboreal	1.424	0.952	<<0.001*	–	Slopes are not equal.	<<0.001*
	Hook-and-pull digging	2.172	0.986	<<0.001*	+		
	Scratch digging	1.952	0.960	<<0.001*	0		
log(DA) ∼ log(IZL)	Arboreal	−0.087	0.119	0.012*	-	Slopes are not equal.	<<0.001*
	Hook-and-pull digging	0.418	0.848	<<0.001*	+		
	Scratch digging	0.132	0.198	0.001*	+		
log(Conn.D) ∼ log(IZL)	Arboreal	−0.767	0.443	<<0.001*	+	Slopes are not equal.	<<0.001*
	Hook-and-pull digging	−3.398	0.854	<<0.001*	0		
	Scratch digging	−1.202	0.920	<<0.001*	+		
log(mass) ∼ log(IZL)	Arboreal	2.904	0.996	<<0.001*	–	Slopes are not equal.	<<0.001*
	Hook-and-pull digging	2.697	0.988	<<0.001*	–		
	Scratch digging	3.530	0.990	<<0.001*	+		

To further quantify the impact of size, ecology, and phylogeny on TBA, we used both pFDA and FDA. We determined ecology groups using the primary locomotor ecology of each genus (Rood 1970; Ramsey 1978; Navarrette and Ortega 2010; Hayssen 2011; Hayssen et al. 2012; Gaudin et al. 2018; Attias et al. 2020), and we determined size class based on the groups from GLS regressions (Table 2). Using the same data as the PGLS, we performed the pFDA using code from Motani and Schmitz 2011 and Smith et al. 2018). This package can only use up to three metrics, so we could not undertake a fully multivariate analysis of our dataset. Therefore, we used three subsets of metrics to complete each analysis: the most size-correlated metrics (Tb.Th, CSA, and Conn.D), the least size-correlated metrics (BV.TV, GC, and DA), and the most phylogenetically-correlated metrics (DA, Tb.Th, and CSA). We chose to use the least size-correlated metrics in our analyses because this model most accurately resolved ecology (Table S4, Fig. S4). For the FDA, we used the mda and nnet R packages to complete the analysis and visualize the results (Venables and Ripley 2002; Leisch and Hornick 2022).

## Results

### Regressions

#### Nonphylogenetic linear regressions

The taxa we examined group in three qualitative size classes: small (*Chlamyphorus, Cyclopes, Tolypeutes*), medium (*Bradypus, Choloepus, Dasypus, Tamandua*), and large (*Myrmecophaga, Priodontes*). These size classes are consistently distinct in our plots (Fig. 5). Because of the strong overlap between ecology and phylogeny in our dataset, we chose to examine relationships between TBA and size using both phylogenetic and nonphylogenetic regressions. When analyzed using nonphylogenetic GLS, all metrics except for DA have a significant correlation with body size. Within this analysis, CSA is the metric most closely correlated with body size (Fig. 5B, Table 2). CSA increases with body size but displays negative allometry with a slope below the isometric expectation. Therefore, CSA is smaller than expected in larger animals. Tb.Th (Fig. 5E) and Conn.D (Fig. 5G) are also closely correlated with size (Table 2). Tb.Th increases as body size increases and has a slight negative allometry, meaning trabeculae are thinner than expected in larger animals. Conn.D decreases as body size increases but is positively allometric. The trabeculae are more connected per unit area in larger animals than expected under isometry despite the overall decreasing trend.

The least size-correlated metrics are BV.TV (Fig. 5C), GC (Fig. 5F), and DA (Fig. 5G) (Table 2). GC decreases as body size increases, but with a low correlation (Table 2), and behaves with slight, but significant, positive allometry, with more bone per unit area than expected as animals get larger. BV.TV increases as body size increases with positive allometry but has the second lowest *R*-squared value of all of the GLS regressions. DA is positively allometric, but is the metric that is least correlated with body size (Fig. 5G, Table 2), and the only metric with a nonsignificant GLS slope (*P* > 0.05). The three size classes are not very distinct in DA, with each class overlapping with the adjacent one(s).

For most metrics, the whole group regression slope lies within the range of the three clade slopes (Tables 2 and 3). GC and DA are the only regressions for which the whole-group slope does not fall within the range of the three clade-specific slopes. The Cingulata and Vermillingua regressions of BV.TV scale with positive allometry, and the Folivora BV.TV regression is isometric (Table 3). The Tb.Th regressions for Cingulata and Vermillingua scale with negative allometry, whereas the Tb.Th regression for Folivora is isometric. GC regressions are positively allometric for Vermillingua and Folivora, but the Cingulata regression is isometric (Table 3). The CSAs of Cingulata and Folivora scale isometrically, whereas the CSA of Vermillingua scales with a slight positive allometry. All of the Cingulata and Vermillingua regressions were significant (*P* < 0.05), whereas only two of the Folivora regressions were significant (GC and CSA). All Vermillingua regressions other than DA had *R*-squared values greater than 0.5. The Cingulata regressions for BV.TV, GC, and DA have *R*-squared values less than 0.5. The only Folivora regression with an *R*-squared value higher than 0.5 is CSA.

#### Equality of slopes test

**Fig. 6:**
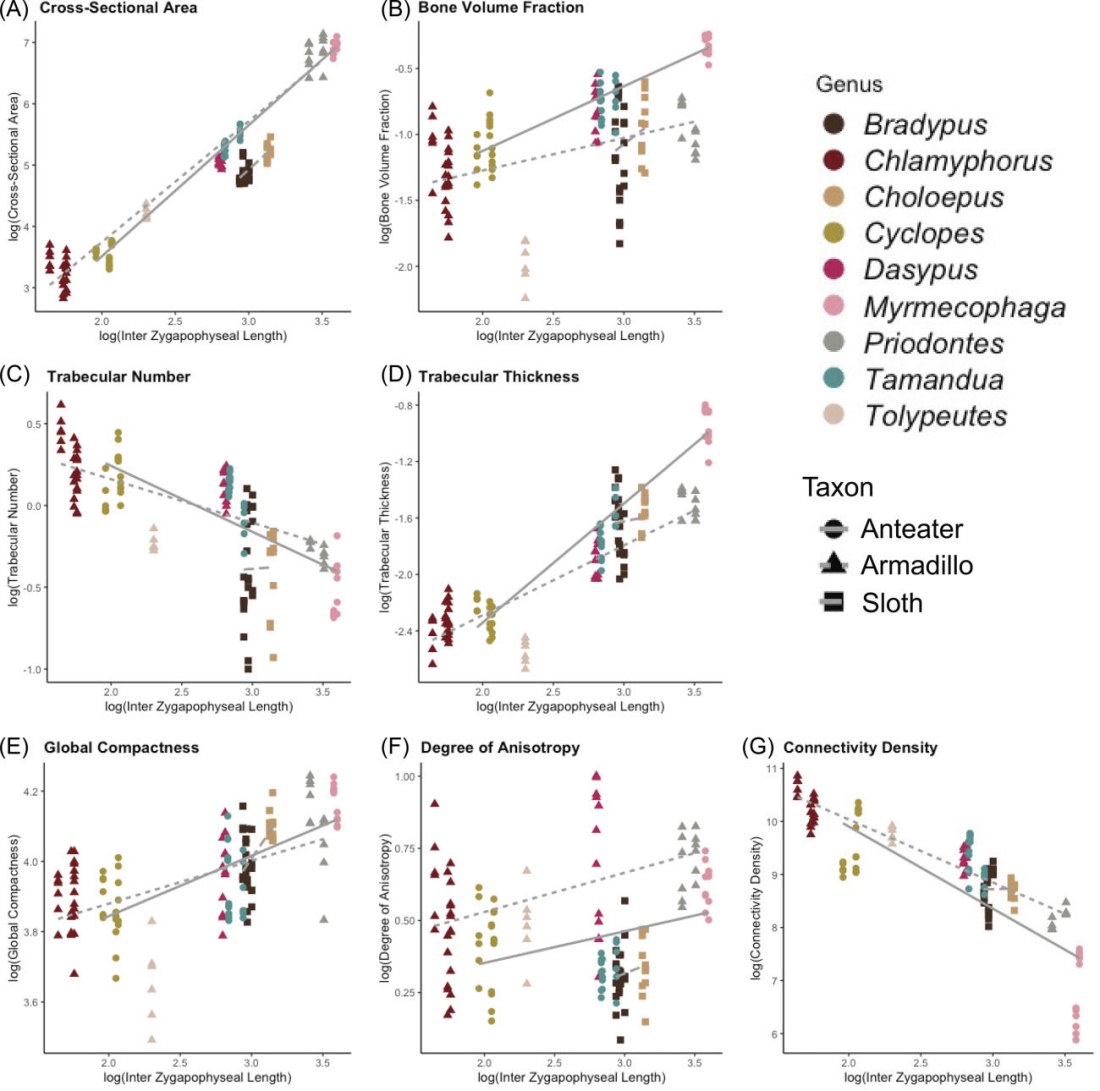
Log10 corrected GLS regressions by taxonomic group. Point shape and line type correspond to taxonomic group, and point color corresponds to genus.

The GLS slopes of the regression lines for each major clade are unequal for all seven metrics. The regression slopes of Cingulata (armadillos) and of Vermillingua (anteaters) are more similar to each other than they are to Folivora (sloths) for most metrics (BV.TV, Tb.N, GC, DA, and Conn.D) (Table 3, Fig. 6). The slopes of Cingulata and Folivora are more similar to each other than to Vermillingua for Tb.Th. The CSA regressions for Vermillingua and Folivora are more similar than they are to Cingulata. The difference in magnitude of slope between Folivora and other clades is largest in the regressions of BV.TV, GC, and Conn.D. The Cingulata and Vermillingua slopes are the most different from one another in the Tb.N, Tb.Th, and Conn.D regressions (Fig. 6).

**Fig. 7:**
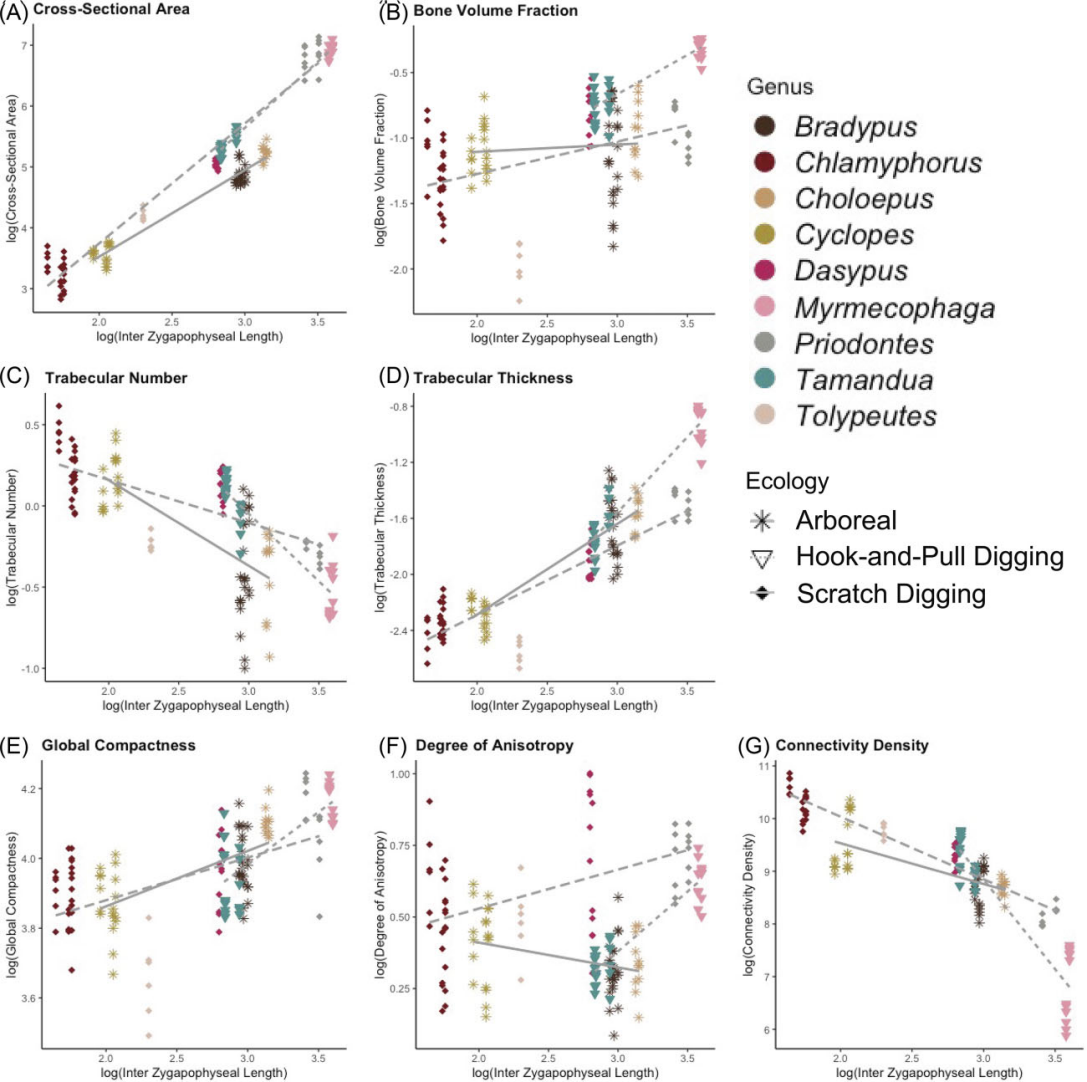
Log10 corrected GLS regressions by ecologic group. Point shape and line type correspond to ecology, and point color corresponds to genus.

The slopes of the three ecological groups are not equal for most metrics (Table 4, Fig. 7). The only metric with equal slopes across ecologies is BV.TV (Table 4), and the only individual regression with a nonsignificant *P*-value is the arboreal BV.TV regression, indicating that the correlation between body size and BV.TV for arboreal xenarthrans is weak. The metric with the largest difference in slopes is Tb.N, indicating that Tb.N correlates with body size differently between ecologies (Table 4). CSA scales differently for each ecological group: with positive allometry for hook-and-pull digging taxa, isometric scaling for scratch digging taxa, and negative allometry for arboreal taxa. The difference in slopes between these groups indicates a possible ecological impact (Table 4).

#### Phylogenetic regressions

Blomberg's *K* ranges from 0.394 to 0.654 for the calculated metrics (Table 5). However, none of the *K*-values are significant (*P* < 0.05). Conn.D has the lowest average *K*-value and the highest *P*-value, indicating that it has the least phylogenetic signal. DA has the highest average *K*-value and the lowest average *P*-value.

**Table 5:** Blomberg's *K*

Metric	Average Blomberg's *K* (range)	Maximum CI value	Average Blomberg's *K P*-value (range)
log(IZL)	0.587 (0.587)	0.00867	0.274 (0.27–0.28)
log(mass)	0.577 (0.577)	0.01899	0.284 (0.27–0.29)
log(BV.TV)	0.461 (0.32–0.60)	0.00831	0.517 (0.26–0.75)
log(Tb.N)	0.412 (0.32–0.59)	0.00985	0.589 (0.28–0.81)
log(Tb.Th)	0.537 (0.46–0.63)	0.00851	0.348 (0.23–0.47)
log(GC)	0.454 (0.37–0.53)	0.01044	0.504 (0.36–0.60)
log(CSA)	0.536 (0.53–0.54)	0.00858	0.330 (0.32–0.34)
log(DA)	0.654 (0.48–0.87)	0.00987	0.236 (0.08–0.43)
log(Conn.D)	0.394 (0.37–0.43)	0.00806	0.639 (0.58–0.68)

The range of PGLS slopes is between 0 and 0.2, with vertebral position having little to no effect on slope estimates. The average slopes of PGLS regressions are very similar to the slopes of the GLS regressions for all metrics (Table 5). The CIs for PGLS slopes were far larger than the GLS CIs, leading to more isometric relationships than in the GLS regressions (Table 2). Due to the larger CIs of the PGLS regressions, many of the phylogenetic slopes imply different scaling relationships than nonphylogenetic slopes (Table 2). The *P*-values of the PGLS regressions of BV.TV and DA and the *P*-value of the GLS regression of DA are the only nonsignificant *P*-values (*P* = 0.2296, *P* = 0.1920, *P* = 0.1460) among the averages of PGLS regressions and GLS regressions. PGLS *P*-values are much less consistent along the vertebral column in a few metrics (BV.TV, Tb.N, and DA) (Table S2).

#### Species average regressions

Species average regressions fall between the individual linear regressions and the phylogenetic regressions for all metrics except for BV.TV (Fig. S3, Table S3). For BV.TV and DA, the species average regression and individual linear regression are far more similar to each other than the phylogenetic regression. The species average regressions for the other five metrics (CSA, Tb.N, Tb.Th, GC, and Conn.D) align much more closely with the phylogenetic regression than with the individual linear regressions (Fig. S3).

### Discriminant function analyses

**Fig. 8:**
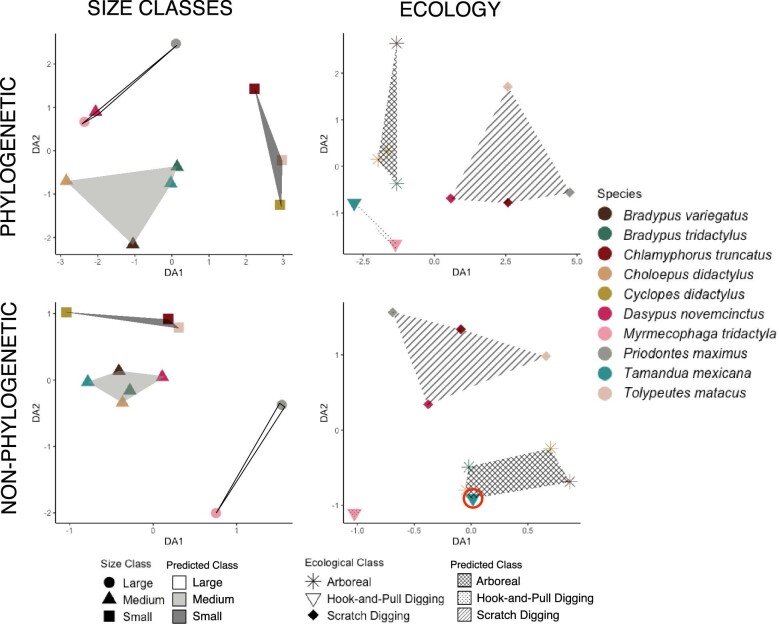
pFDA and FDA graphs of the first position for analysis: Point color corresponds to species. Point shape corresponds to true class. Red circles indicate incorrect categorization. Predicted classes are depicted by convex hulls with fill corresponding to predicted class.

Size classes are consistently and accurately resolved for both FDA and pFDA (Tables 6, S4, and S5, Figs. 8, S4, and S6), but the FDA is more accurate (0.97 correct classification rate) than pFDA (0.85 correct classification rate) (Table 6, S4). Vertebral position has little effect on the accuracy of FDA or pFDA. The medium size class is always accurately resolved for the FDA (Table 6). The large size class is consistently accurately resolved for the pFDA, whereas the medium and small size classes almost always have one taxon miscategorized (Table 6). The miscategorized taxa are most often *Cyclopes didactylus* and *Dasypus novemcinctus* (Fig. S6).

**Table 6:** Discriminant function analyses

Group	Position for analysis	Accuracy
Size class	ps1	1.0
	ps2	1.0
	ps3	1.0
	ps4	1.0
	ps5	1.0
	ps6	0.8
	All positions	0.97
Phylogenetic size class	ps1	0.9
	ps2	0.8
	ps3	1.0
	ps4	0.8
	ps5	0.8
	ps6	0.8
	All positions	0.85
Ecology	ps1	0.9
	ps2	0.9
	ps3	0.9
	ps4	0.9
	ps5	0.9
	ps6	0.9
	All positions	0.9
Phylogenetic ecology	ps1	1.0
	ps2	0.9
	ps3	0.7
	ps4	1.0
	ps5	0.9
	ps6	0.6
	All positions	0.85

Ecology is categorized with similar accuracy as size classes (Table 6). For FDA there is always one taxon miscategorized (either *C. didactylus* or *Tamandua mexicana*) (Table 6, Figs. 8, S5). *Cyclopes didactylus* is miscategorized most often (either as hook-and-pull digging [ps5 and ps6] or as scratch digging [ps3] [Fig. S5]). *Bradypus tridactylus* is miscategorized as hook-and-pull digging twice. *Choloepus didactylus, Bradypus variegatus, P. maxiumus*, and *C. truncatus* are all miscategorized once (Fig. S4).

The pFDA is less accurate than FDA, with correct classification rates ranging from 0.6 (sixth vertebral position) to 1.0 (first and fourth vertebral position) and a total accuracy of 0.85 (Table 6, Figs. 8, S7). Hook-and-pull digging taxa are always correctly categorized, whereas arboreal taxa are correctly categorized the least often (Table 5). Scratch-digging taxa (*C. truncatus* and *D. novemcinctus*) are only miscategorized when using the last vertebral position (Table 6, S5).

## Discussion

In this work, we examined the vertebral bone microstructure of xenarthran mammals to untangle the impacts of body size, ecology, and phylogeny on their TBA. All metrics measured are influenced by both body size and ecology, but the magnitude of the effect differs for each metric. CSA and Tb.Th have the highest body size signal (Table 2), whereas BV.TV and DA have the lowest size signal (Table 2). BV.TV, Tb.N, GC, and Conn.D are positively allometric, whereas Tb.Th and CSA are negatively allometric (Table 2). These patterns are consistent with other work examining the relationship between body size and TBA (Doube et al. 2011; Plasse et al. 2019; Saers et al. 2019).

Ecological groups have significantly different regression slopes for all metrics except BV.TV using nonphylogenetic methods. These groups are also consistently resolved using nonphylogenetic discriminant function analyses (0.9 accuracy) (Table 6). They are less consistently resolved with PFDA; there is a high average accuracy (0.85 accuracy), but this varies much more along the vertebral column (Table 6). Because of the overlap between ecology and phylogeny, it is not possible to entirely disentangle their impacts, and current phylogenetic comparative methods may struggle to differentiate between ecological and phylogenetic impacts (Uyeda et al. 2018, see Case Study 3 specifically). The differences between the results of the phylogenetic analyses and the nonphylogenetic analyses indicate that there is likely an impact of phylogeny on this system, but cleanly isolating this component of variation is complicated by the overlap between ecology and phylogeny (Uyeda et al. 2018).

### Impacts of phylogeny

We attempted to measure phylogenetic impact by comparing phylogenetic and nonphylogenetic statistical methods, but the close correspondence between phylogeny and ecology in extant xenarthrans means we likely cannot completely disentangle the influence of phylogeny and ecology in this group (Fig. 1). We treated phylogenetic comparative methods as one extreme because they assume that the covariance of the model is proportional to branch lengths on the tree (i.e., similarity between closely related organisms is due to phylogeny rather than convergence) and nonphylogenetic methods as the other extreme because they assume that the residual error of the model is independent (i.e., all differences are due to ecological convergence) (Revell 2010).

Because PGLS does not allow for multiple specimens from one species, we attempted to measure the impact of using species means in the phylogenetic regressions by comparing the three regression methods we used: GLS regression, GLS regression with species means, and phylogenetic regression (also using species means) (Fig. S3). For most metrics, the phylogenetic slope and species average slope align, potentially indicating that the perceived phylogenetic signal is actually caused by using the species averages required for phylogenetic methods. However, the PGLS slopes for DA and BV.TV are notably different from the other two slopes that are very close (Fig. S3). This indicates that there is likely a phylogenetic signal in both DA and BV.TV.

Application of phylogenetic comparative methods has some impact on most metrics and often implies a moderate amount of phylogenetic signal, especially for DA (Blomberg's *K* = 0.654), Tb.Th (Blomberg's *K* = 0.537), and CSA (Blomberg's *K* = 0.536). However, it is difficult to differentiate between ecological and phylogenetic signals due to the overlap between phylogeny and ecology (Fig. 1). Because all scratch diggers are within Cingulata, all hook-and-pull diggers are within Vermillingua, and all extant Folivora are arboreal, any perceived phylogenetic signal likely has an ecological component, and any perceived ecological signal likely includes a phylogenetic effect.

The scaling patterns for many of the phylogenetic regressions are isometric, whereas nonphylogenetic regressions are allometric (Table 2). This occurs for BV.TV, Tb.Th, and CSA. The average CIs for all PGLS regressions tend to be far larger than for the GLS regressions (Table 2). The larger CIs for the PGLS regressions are likely what cause the difference in scaling relationships. The size of the CIs is potentially due to the smaller sample size of species means used in phylogenetic regressions or another manifestation of the strong correlation between phylogeny and ecology. The difference between phylogenetic and nonphylogenetic regressions implies that there is some phylogenetic signal and some ecological signal in this system.

Although there has been no work explicitly investigating the relationship between phylogeny and TBA, it has been secondarily investigated in limb bones, and mostly in primates (Ryan and Shaw 2013; Tsegai et al. 2013; Kivell 2016; Plasse et al. 2019). BV.TV, Tb.N, trabecular spacing, Conn.D, and DA have all previously been found to have phylogenetic signal, but this is not consistent among groups or among the bones examined. These findings somewhat align with ours: BV.TV has some phylogenetic signal when comparing regression methods; DA has some phylogenetic signal when using Blomberg's *K* (Tables 2 and 5). However, no previous work has found any phylogenetic signal in Tb.Th or CSA, and CSA has not been examined in any of this previous work (Ryan and Shaw 2013; Tsegai et al. 2013; Kivell 2016; Plasse et al. 2019).

We find that there is some phylogenetic signal in the TBA of xenarthran vertebrae both through comparing regressions methods and through calculating Blomberg's *K* (Fig. S3, Table 5). Our findings somewhat align with the previous work, but this work has been very limited (Ryan and Shaw 2013; Tsegai et al. 2013; Kivell 2016; Plasse et al. 2019). It is unclear how much of the detected phylogenetic signal is due to phylogeny and how much is due to ecology, but there is clearly both ecological and phylogenetic signal in this system.

### Measurements of combined bone density

Three of the most interconnected metrics we measured are CSA, BV.TV, and GC. CSA is the metric most closely related to body size, which makes sense given that CSA measures the area of bone in a single image slice of a vertebra (Amson 2019). However, when using nonphylogenetic regressions, CSA is negatively allometric, indicating that the cross sections of the vertebrae of larger xenarthrans are smaller than expected, and when using phylogenetic methods, CSA is isometric. The negative allometry of CSA is especially surprising given the positive allometry of BV.TV and GC. BV.TV and GC measure the same property over different volumes of interest. BV.TV is measured over a spherical volume in the middle of the centrum, whereas GC is measured over the entire vertebra, including the cortical bone and the neural arch (Amson 2019). The positive allometry of BV.TV and GC indicates that there is less space between individual trabeculae in larger individuals than would be expected in an isometric relationship (Doube et al. 2011; Barak et al. 2013), but the low *R*-squared values for BV.TV and GC suggest that the body size effect in these metrics is weak. In contrast, CSA has the highest *R*-squared value (Table 2). This is potentially caused by the upper and lower limits of Tb.Th and the range of body size within our sample (Lozupone and Favia 1990; Mullender and Huiskes 1995; Jones et al. 2009; Barak et al. 2013; Ryan and Shaw 2013): larger animals have more (Tb.N), relatively thinner (Tb.Th), trabeculae, whereas smaller animals have fewer, relatively thicker trabeculae, leading to positive allometry of measures of combined bone density (Table 2).

**Fig. 9:**
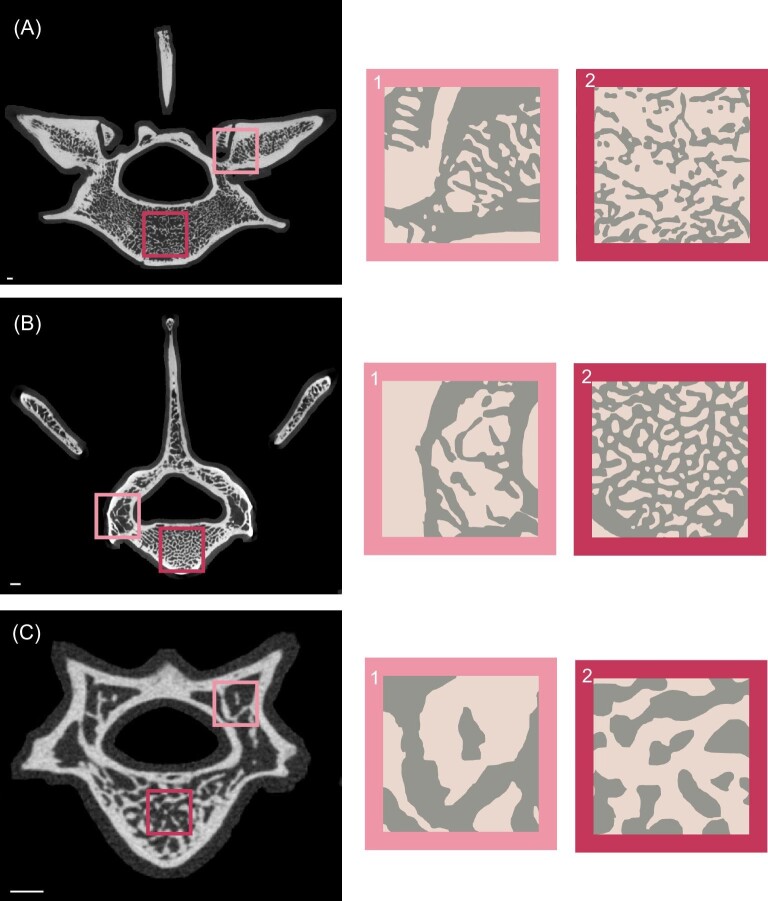
Representative μCT slices from *P. maximus* (FMNH 72913), *D. novemcinctus* (FMNH 39307), and *C. truncatus* (FMNH 39468) showing the difference between GC in vertebral centra and neural arch.

The negative allometry of CSA and the positive allometry of BV.TV and GC are potentially caused by the difference between the density of trabeculae in the vertebral centrum and the density of trabeculae in the neural arch (Fig. 9). The density of trabeculae in the neural arch is qualitatively lower than that in the vertebral centrum. This qualitative difference is consistent throughout the entire size range of our dataset from *C. truncatus* to *Priodontes maximus* (Fig. 9A–C). This makes some functional sense because the centrum likely experiences more force than the neural arch (Smit et al. 1997) and would therefore adaptively benefit from the increased strength associated with increased BV.TV (Ulrich et al. 1999).

### Impact of body size variation on scaling

The scaling relationships of TBA in this system are complicated further by the distribution of body mass within extant xenarthrans. We optimized body mass on a xenarthran phylogeny (Figs. S1 and S2). Both the largest (*P. maximus*, mass = 40641.09 g) and the smallest (*C. truncatus*, mass = 85.53 g) xenarthrans are within Cingulata (Table 1), and there is a similarly large body mass disparity in Vermillingua between *M. tridactyla* (mass = 29531.83 g) and *Cy. didactylus* (mass = 263.95 g) (Table 1). However, this range of body mass is missing in extant Folivora. Because of the large disparity found in two of the three xenarthran groups, there was no clear phylogenetic signal for body mass. If extinct xenarthrans were included in this phylogeny, then these signals might shift, especially due to the large number of large-bodied fossil sloths (Fariña and Blanco 1996; Per Christiansen and Fariña 2003; Brandoni et al. 2010; Amson et al. 2015). The lack of size range in extant Folivora potentially impacts the lack of scaling relationships within sloths.

### Patterns within Folivora

There are only six extant species of sloths, and three of those species are represented by wild specimens in the FMNH mammalogy collections. Because of this limited diversity and specimen scarcity, the sample of Folivora within our data set includes three species that all have very similar body sizes (*B. tridactylus* = 4136.36 g, *B. variegatus* = 4375.80 g, *Ch. didactylus* = 6646.50 g) (Table 1). Therefore, parsing the impact of body size, ecology, and phylogeny within this group is especially difficult.

In all taxonomic equality of slope tests, Folivora has very different slopes, *R*-squared values, and *P*-values from the other clades. The only metric with a significant correlation with body size for sloths is CSA, and most metrics have *R*-squared less than 0.05 (Table 3). It is unclear whether this is due to the small body size range or the suspensory ecology of extant sloths, which results in unique loading of the vertebral column in sloths (Nyakatura and Fischer 2010; Olson et al. 2018; Alfieri et al. 2021).

This problem could potentially be solved by including fossil taxa in our analysis. The impressive diversity of extinct sloths could greatly increase the range of sizes and ecologies represented in the clade, allowing us to better examine the relationship between TBA, body size, and ecology within this group. There has been some examination of extinct sloth TBA, but not enough to provide any insight into how scaling or ecology impact the TBA of their vertebrae; these papers did not include sufficient data to use in our analyses (Fariña and Blanco 1996; Per Christiansen and Fariña 2003; Brandoni et al. 2010; Amson et al. 2015; Montañez-Rivera et al. 2018; Alfieri et al. 2021). In particular, it would provide an excellent test of whether the unusual TBA properties of extant sloths is a characteristic of all folivorans, or one that stems from a suspensory lifestyle (Amson et al. 2017; Montañez-Rivera et al. 2018; Alfieri et al. 2021). Additionally, a data set including both extant and fossil xenarthrans could encompass the majority of terrestrial mammalian size disparity. This would allow us to examine more completely whether scaling patterns found when examining all of Mammalia are also found within smaller clades.

### Tolypeutes matacus

**Fig. 10:**
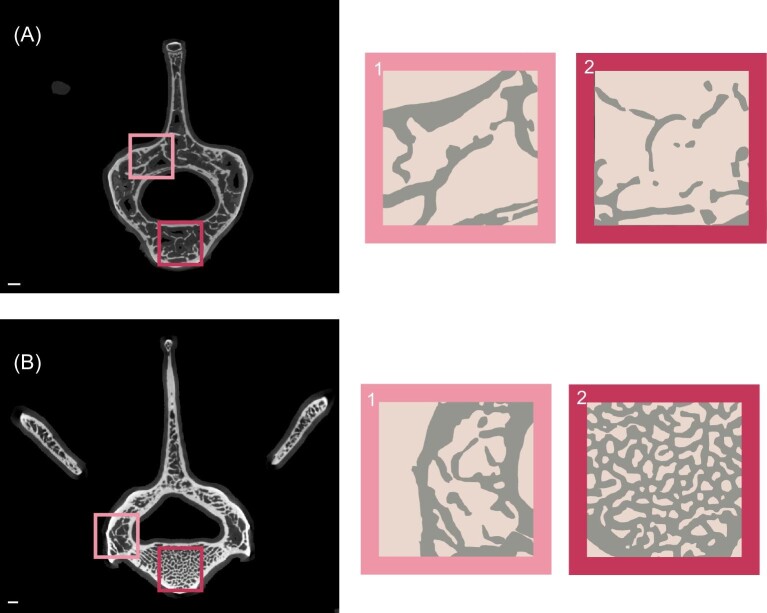
Representative μCT slices from (A) *T. matacus* (FMNH 28342) and (B) *D. novemcinctus* (FMNH 39307) demonstrating the low trabecular density of *Tolypeutes* as compared to *Dasypus*.

***Tolypeutes matacus*** (southern three-banded armadillo) falls far below the whole dataset regression line in four (BV.TV, Tb.N, Tb.Th, and GC) of the seven regressions (Fig. 5C–F). These metrics all tell us about the amount of trabecular bone within a VOI, meaning that *T. matacus* has fewer, thinner trabeculae than would be expected given its size (Fig. 10). *Tolypeutes* is the only armadillo that can fully roll into a ball, and it has previously been suggested that *T. matacus* is the least fossorial armadillo and does not dig at all (Sanborn 1930; Guimaraes 1997; Eisenberg and Redford 1999; Smith 2007; Wetzel et al. 2008). This could make functional sense as a less fossorial lifestyle requires less force transmission through the vertebral column during high-force digging activities (Hildebrand 1985; Gaudin and Biewener 1992; Clerici et al. 2018) and might be associated with less robust TBA, but more recent studies have found that they can and do dig their own burrows (Galliari et al. 2010; Attias et al. 2016). Nevertheless, our results suggest that some aspect of their ecology may lead to unusually light vertebral loading. Other unique aspects of morphology related to the rolling capability of *T. matacus* may be acting in a compensatory fashion, withstanding some of the load that would usually be borne by the vertebral centrum. More research on the anatomy of the epaxial musculature and vertebral function of *T. matacus* is required to fully understand these results.

## Conclusion

TBA in xenarthran vertebrae is impacted by body size, phylogeny, and ecology. BV.TV, Tb.N, and DA have the strongest ecological and phylogenetic signals, whereas CSA, Tb.Th, and Conn.D are more impacted by body size. Discriminant function analysis consistently resolves size classes using trabecular bone measurements, indicating that TBA overall is closely linked to body size.

Larger xenarthran vertebrae have more bone versus total CSA than expected under isometry. The trabeculae are thinner than expected, and there are relatively more of them. Smaller xenarthran vertebrae have fewer, thicker trabeculae than expected under isometry. The relationship between body size and trabecular bone is more consistent in digging taxa than in arboreal taxa. Folivora has no consistent patterns between body size and TBA, which is possibly due to the limited size range in extant Folivora or may be related to the suspensory ecology of all extant sloths. *Tolypeutes matacus* falls below the regression lines for many metrics, potentially due to its ability to roll into a ball, but more comparative anatomical information is required to understand the functional morphology of this unusual species.

Any ecological or phylogenetic impact on xenarthran trabecular bone is complicated by the similarity between ecological groups and taxonomic groups in this clade. All armadillos are scratch digging, all extant sloths are arboreal, and all hook-and-pull diggers are anteaters. This issue is reflected by the fact that ecological pFDA analysis is less accurate and less consistent than the ecological FDA, suggesting that ecological and phylogenetic covariance are not clearly distinguished by the pFDA. Further studies including fossil xenarthrans (ground sloths) likely would help to separate the impact of ecology from the impact of phylogeny by expanding the ranges of ecologies and body sizes represented, especially in Folivora.

## Supplementary Material

obad002_Supplemental_File

## Data Availability

Grayscale and binary VOI image stacks will be made publicly available through Morphosource. Full reconstructed image stacks for each specimen will be deposited at the Field Museum of Natural History and linked to original specimen records through EMu. These image stacks will be available upon request.
