# Supplementary material for: From Fairies to Giants: Untangling the Effect of Body Size, Phylogeny, and Ecology on Vertebral Bone Microstructure of Xenarthran Mammals
Source: Integr Org Biol. 2023 Jan 16;5(1):obad002. doi: 10.1093/iob/obad002 (PMC9949600; doi:10.1093/iob/obad002)
Supplement: obad002_Supplemental_File [file obad002_supplemental_file.docx]

FIGURE S1:


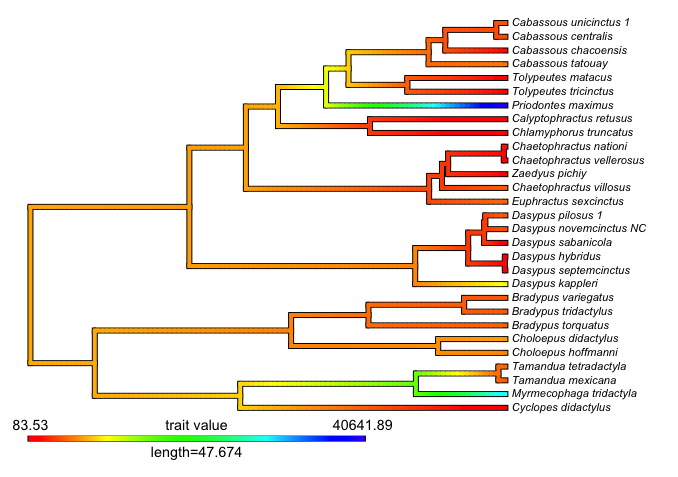


Xenarthran Phylogeny with average body mass.

FIGURE S2:


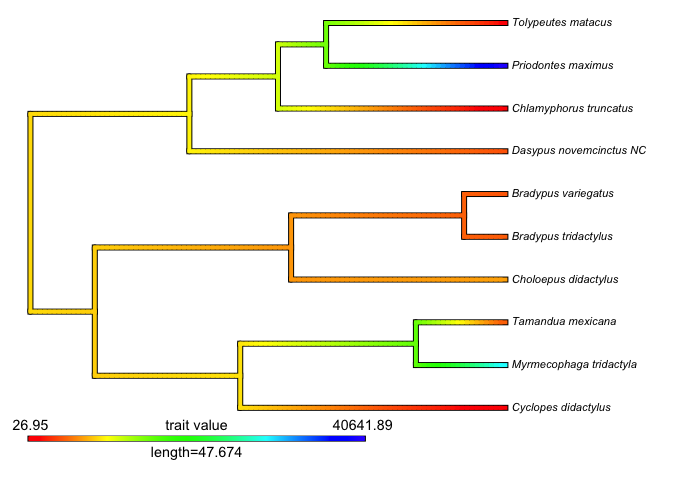


Xenarthran Phylogeny including taxa sampled with body mass signal.

TABLE S1: Inter-Zygapophyseal Length Regressions

| Position for Analysis | Slope | p-value (ɑ < 0.05) | R-squared |
| --- | --- | --- | --- |
| ps1 | 2.9486 | << 0.001 * | 0.9579 |
| ps2 | 3.1895 | << 0.001 * | 0.9654 |
| ps3 | 3.2037 | << 0.001 * | 0.9779 |
| ps4 | 3.2491 | << 0.001 * | 0.9615 |
| ps5 | 3.2948 | << 0.001 * | 0.9585 |
| ps6 | 2.9855 | << 0.001 * | 0.9685 |

FIGURE S3: Regression Method Comparison


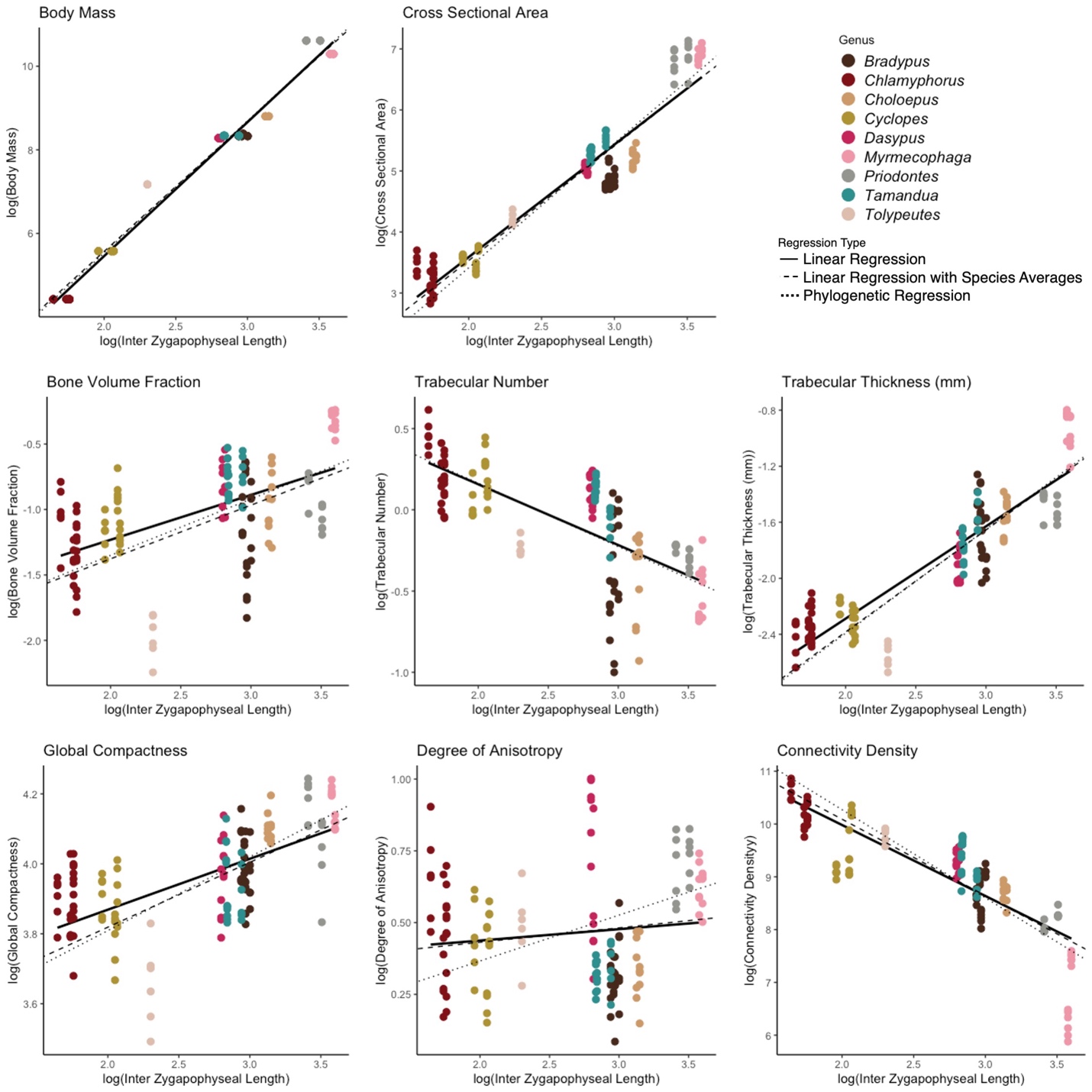


Log_10_ corrected regressions using three methods: GLS regression with individual data, GLS regression with species averages, and PGLS regressions with species averages. Line type corresponds to regression method, and point color corresponds to genus.

TABLE S2: Phylogenetic regression

| Formula | Position for Analysis | Slope | Lower Confidence Interval | Upper Confidence Interval | Slope p-value (ɑ < 0.05) |
| --- | --- | --- | --- | --- | --- |
| log(BV.TV) ~ log(IZL) | ps1 | 0.3498 | -0.445 | 1.145 | 0.340 |
|  | ps2 | 0.4345 | -0.077 | 0.946 | 0.086 |
|  | ps3 | 0.5030 | -0.121 | 1.127 | 0.100 |
|  | ps4 | 0.4559 | -0.061 | 0.973 | 0.077 |
|  | ps5 | 0.4283 | -0.267 | 1.123 | 0.193 |
|  | ps6 | 0.4083 | -0.349 | 1.166 | 0.249 |
| log(Tb.N) ~ log(IZL) | ps1 | -0.4369 | -0.820 | -0.054 | 0.030 * |
|  | ps2 | -0.4145 | -0.729 | -0.100 | 0.016 * |
|  | ps3 | -0.3702 | -0.701 | -0.039 | 0.033 * |
|  | ps4 | -0.4165 | -0.701 | -0.132 | 0.010 * |
|  | ps5 | -0.3792 | -0.942 | 0.184 | 0.159 |
|  | ps6 | -0.3244 | -0.965 | 0.316 | 0.276 |
| log(Tb.Th) ~ log(IZL) | ps1 | 0.6867 | 0.176 | 1.198 | 0.015 * |
|  | ps2 | 0.7664 | 0.410 | 1.122 | 0.001 * |
|  | ps3 | 0.7304 | 0.383 | 1.078 | 0.001 * |
|  | ps4 | 0.7745 | 0.458 | 1.091 | << 0.001 * |
|  | ps5 | 0.7619 | 0.516 | 1.008 | << 0.001 * |
|  | ps6 | 0.7334 | 0.495 | 0.972 | << 0.001 * |
| log(GC) ~ log(IZL) | ps1 | 0.1670 | 0.008 | 0.326 | 0.042 * |
|  | ps2 | 0.1955 | 0.006 | 0.385 | 0.045 * |
|  | ps3 | 0.1973 | 0.063 | 0.332 | 0.010 * |
|  | ps4 | 0.2632 | 0.058 | 0.469 | 0.018 * |
|  | ps5 | 0.1978 | 0.040 | 0.356 | 0.020 * |
|  | ps6 | 0.2434 | 0.057 | 0.430 | 0.017 * |
| log(CSA) ~ log(IZL) | ps1 | 1.9497 | 1.669 | 2.231 | << 0.001 * |
|  | ps2 | 2.0555 | 1.758 | 2.353 | << 0.001 * |
|  | ps3 | 2.0306 | 1.642 | 2.419 | << 0.001 * |
|  | ps4 | 2.0352 | 1.638 | 2.433 | << 0.001 * |
|  | ps5 | 2.0554 | 1.680 | 2.430 | << 0.001 * |
|  | ps6 | 2.1157 | 1.762 | 2.468 | << 0.001 * |
| log(DA) ~ log(IZL) | ps1 | 0.1059 | -0.061 | 0.272 | 0.181 |
|  | ps2 | 0.1572 | 0.003 | 0.312 | 0.047 * |
|  | ps3 | 0.2220 | -0.016 | 0.460 | 0.063 * |
|  | ps4 | 0.1663 | 0.033 | 0.300 | 0.021 * |
|  | ps5 | 0.1414 | -0.111 | 0.393 | 0.232 |
|  | ps6 | 0.1663 | -0.096 | 0.425 | 0.177 |
| log(Conn.D) ~ log(IZL) | ps1 | -1.6508 | -2.350 | -0.951 | << 0.001 * |
|  | ps2 | -1.6578 | -2.408 | -0.908 | << 0.001 * |
|  | ps3 | -1.6084 | -2.421 | -0.796 | 0.002 * |
|  | ps4 | -1.7344 | -2.597 | -0.872 | 0.002 * |
|  | ps5 | -1.7465 | -2.753 | -0.740 | 0.004 * |
|  | ps6 | -1.6241 | -2.546 | -0.703 | 0.004 * |

TABLE S3: Species Average Regressions

| Formula | Isometric Slope | Species Average GLS Slope | Species Average Confidence Interval | Species Average Slope p-value (ɑ < 0.05) | Species Average R-squared value | Species Average Allometry |
| --- | --- | --- | --- | --- | --- | --- |
| log(BV.TV) ~ log(IZL) | 0 | 0.41 | 0.23 – 0.59 | << 0.001 * | 0.262 | + |
| log(Tb.N) ~ log(IZL) | -1 | -0.37 | -0.47 – -0.26 | << 0.001 * | 0.459 | + |
| log(Tb.Th) ~ log(IZL) | 1 | 0.73 | 0.63 – 0.83 | << 0.001 * | 0.795 | - |
| log(GC) ~ log(IZL) | 0 | 0.19 | 0.13 – 0.24 | << 0.001 * | 0.480 | + |
| log(CSA) ~ log(IZL) | 2 | 1.88 | 1.70 – 2.06 | << 0.001 * | 0.882 | 0 |
| log(DA) ~ log(IZL) | 0 | 0.05 | -0.03 – 0.13 | 0.214 | 0.807 | 0 |
| log(Conn.D) ~ log(IZL) | -3 | -1.45 | -1.63 – -1.26 | << 0.001 * | 0.026 | + |
| log(mass) ~ log(IZL) | 3 | 3.11 | 2.96 – 3.27 | << 0.001 * | 0.966 | 0 |

Table S4: FDA and pFDA Accuracy

| Metrics | Groups | Phylogenetic Correction | Position for Analysis | Accuracy (* all accurate) |
| --- | --- | --- | --- | --- |
| Most Size Correlated (Tb.Th, CSA, and Conn.D) | Size | no | ps1 | 1.0 * |
|  |  |  | ps2 | 1.0 * |
|  |  |  | ps3 | 1.0 * |
|  |  |  | ps4 | 1.0 * |
|  |  |  | ps5 | 1.0 * |
|  |  |  | ps6 | 1.0 * |
|  |  |  | all positions | 1.0 * |
|  |  | yes | ps1 | 1.0 * |
|  |  |  | ps2 | 1.0 * |
|  |  |  | ps3 | 1.0 * |
|  |  |  | ps4 | 1.0 * |
|  |  |  | ps5 | 1.0 * |
|  |  |  | ps6 | 1.0 * |
|  |  |  | all positions | 1.0 * |
|  | Ecology | no | ps1 | 0.8 |
|  |  |  | ps2 | 0.8 |
|  |  |  | ps3 | 0.8 |
|  |  |  | ps4 | 0.8 |
|  |  |  | ps5 | 0.9 |
|  |  |  | ps6 | 1.0 * |
|  |  |  | all positions | 0.85 |
|  |  | yes | ps1 | 0.8 |
|  |  |  | ps2 | 0.5 |
|  |  |  | ps3 | 0.6 |
|  |  |  | ps4 | 0.6 |
|  |  |  | ps5 | 0.8 |
|  |  |  | ps6 | 0.7 |
|  |  |  | all positions | 0.67 |
| Least Size Correlated (BV.TV, GC, and DA) | Size | no | ps1 | 1.0 * |
|  |  |  | ps2 | 1.0 * |
|  |  |  | ps3 | 1.0 * |
|  |  |  | ps4 | 1.0 * |
|  |  |  | ps5 | 1.0 * |
|  |  |  | ps6 | 0.8 |
|  |  |  | all positions | 0.97 |
|  |  | yes | ps1 | 0.9 |
|  |  |  | ps2 | 0.8 |
|  |  |  | ps3 | 1.0 * |
|  |  |  | ps4 | 0.8 |
|  |  |  | ps5 | 0.8 |
|  |  |  | ps6 | 0.8 |
|  |  |  | all positions | 0.85 |
|  | Ecology | no | ps1 | 0.9 |
|  |  |  | ps2 | 0.9 |
|  |  |  | ps3 | 0.9 |
|  |  |  | ps4 | 0.9 |
|  |  |  | ps5 | 0.9 |
|  |  |  | ps6 | 0.9 |
|  |  |  | all positions | 0.9 |
|  |  | yes | ps1 | 1.0 * |
|  |  |  | ps2 | 0.9 |
|  |  |  | ps3 | 0.7 |
|  |  |  | ps4 | 1.0 * |
|  |  |  | ps5 | 0.9 |
|  |  |  | ps6 | 0.6 |
|  |  |  | all positions | 0.85 |
| Most Phylogenetically Correlated  (DA, Tb.Th, CSA) | Size | no | ps1 | 1.0 * |
|  |  |  | ps2 | 1.0 * |
|  |  |  | ps3 | 1.0 * |
|  |  |  | ps4 | 1.0 * |
|  |  |  | ps5 | 1.0 * |
|  |  |  | ps6 | 1.0 * |
|  |  |  | all positions | 1.0 * |
|  |  | yes | ps1 | 1.0 * |
|  |  |  | ps2 | 1.0 * |
|  |  |  | ps3 | 1.0 * |
|  |  |  | ps4 | 1.0 * |
|  |  |  | ps5 | 1.0 * |
|  |  |  | ps6 | 1.0 * |
|  |  |  | all positions | 1.0 * |
|  | Ecology | no | ps1 | 0.9 |
|  |  |  | ps2 | 0.9 |
|  |  |  | ps3 | 0.8 |
|  |  |  | ps4 | 1.0 * |
|  |  |  | ps5 | 0.9 |
|  |  |  | ps6 | 1.0 * |
|  |  |  | all positions | 0.92 |
|  |  | yes | ps1 | 0.7 |
|  |  |  | ps2 | 0.8 |
|  |  |  | ps3 | 0.7 |
|  |  |  | ps4 | 0.9 |
|  |  |  | ps5 | 0.8 |
|  |  |  | ps6 | 0.7 |
|  |  |  | all positions | 0.77 |

FIGURE S4: FDA Accuracy


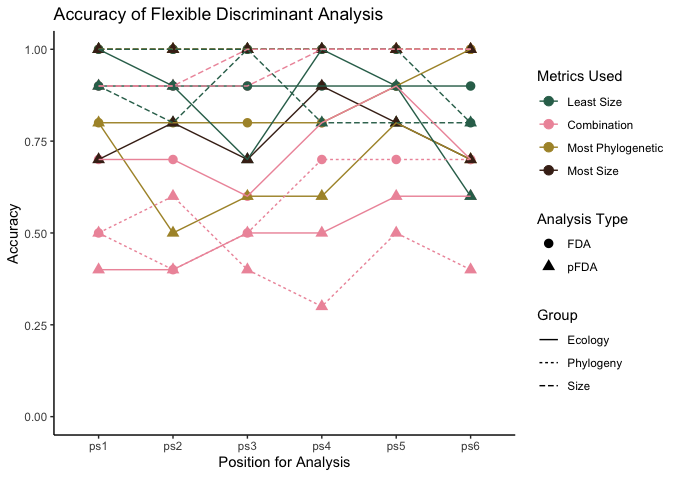


Accuracy of phylogenetic discriminant function analysis and flexible discriminant analysis. Line color corresponds to metrics used for the analysis. Line type corresponds to groups analyzed. Point shape corresponds to phylogenetic correction.

TABLE S5: Discriminant Function Analysis Grouped by Size Class

| Group | Position for Analysis | Accuracy | Large Group Accuracy | Medium Group Accuracy | Small Group Accuracy |
| --- | --- | --- | --- | --- | --- |
| Size Class | ps1 | 1.0 | 1.0 | 1.0 | 1.0 |
|  | ps2 | 1.0 | 1.0 | 1.0 | 1.0 |
|  | ps3 | 1.0 | 1.0 | 1.0 | 1.0 |
|  | ps4 | 1.0 | 1.0 | 1.0 | 1.0 |
|  | ps5 | 1.0 | 1.0 | 1.0 | 1.0 |
|  | ps6 | 0.8 | 0.5 | 1.0 | 0.67 |
| Phylogenetic Size Class | ps1 | 0.9 | 1.0 | 0.8 | 1.0 |
|  | ps2 | 0.8 | 1.0 | 0.8 | 0.67 |
|  | ps3 | 1.0 | 1.0 | 1.0 | 1.0 |
|  | ps4 | 0.8 | 1.0 | 0.8 | 0.67 |
|  | ps5 | 0.8 | 1.0 | 0.8 | 0.67 |
|  | ps6 | 0.8 | 1.0 | 0.8 | 0.67 |

TABLS S6: Discriminant Function Analysis Grouped by Ecology

| Group | Position for Analysis | Accuracy | Scratch Digging Group Accuracy | Hook-and-Pull Digging Group Accuracy | Arboreal Group Accuracy |
| --- | --- | --- | --- | --- | --- |
| Ecology | ps1 | 0.9 | 1.0 | 0.5 | 1.0 |
|  | ps2 | 0.9 | 1.0 | 1.0 | 0.75 |
|  | ps3 | 0.9 | 1.0 | 0.5 | 1.0 |
|  | ps4 | 0.9 | 1.0 | 0.5 | 1.0 |
|  | ps5 | 0.9 | 1.0 | 1.0 | 0.75 |
|  | ps6 | 0.9 | 1.0 | 1.0 | 0.75 |
| Phylogenetic Ecology | ps1 | 1.0 | 1.0 | 1.0 | 1.0 |
|  | ps2 | 0.9 | 1.0 | 1.0 | 0.75 |
|  | ps3 | 0.7 | 1.0 | 1.0 | 0.5 |
|  | ps4 | 1.0 | 1.0 | 1.0 | 1.0 |
|  | ps5 | 0.9 | 1.0 | 1.0 | 0.75 |
|  | ps6 | 0.6 | 0.5 | 1.0 | 0.5 |

FIGURE S5: Flexible Discriminant Analysis by Ecology

Flexible discriminant analysis by ecology for all vertebral positions. Point color corresponds to species. Point shape corresponds to true class. Predicted classes are depicted by convex hulls with fill corresponding to predicted class. Red circles represent miscategorized taxa.

FIGURE S6: Flexible Discriminant Analysis by Size

Flexible discriminant analysis by size for all vertebral positions. Point color corresponds to species. Point shape corresponds to true class. Predicted classes are depicted by convex hulls with fill corresponding to predicted class. Red circles represent miscategorized taxa.

FIGURE S7: Phylogenetic Flexible Discriminant Analysis by Ecology Phylogenetic flexible discriminant analysis by ecology for all vertebral positions. Point color corresponds to species. Point shape corresponds to true class. Predicted classes are depicted by convex hulls with fill corresponding to predicted class. Red circles represent miscategorized taxa.

FIGURE S8: Phylogenetic Flexible Discriminant Analysis by Size

 Phylogenetic flexible discriminant analysis by size for all vertebral positions. Point color corresponds to species. Point shape corresponds to true class. Predicted classes are depicted by convex hulls with fill corresponding to predicted class. Red circles represent miscategorized taxa.
